# ‘Background to clinical guidelines in cancer’: SOR, a programmatic approach to guideline development and aftercare

**DOI:** 10.1054/bjoc.2001.1755

**Published:** 2001-05

**Authors:** G Browman

**Affiliations:** Hamilton Regional Cancer Centre and McMaster University, Hamilton, Canada


British Journal of Cancer (2001) 84 (Supplement 2), 1–3
© 2001 FNCLCC
doi: 10.1054/ bjoc.2001.1755, available online at http://www.idealibrary.com on IDEAL®

### 'Background to clinical guidelines in cancer': SOR, a programmatic approach to guideline development and aftercare

**G Browman**

Hamilton Regional Cancer Centre and McMaster University, Hamilton, Canada

The SOR (Standards, Options, Recommendations) clinical practice guideline program of the FNCLCC (French National Federation of Cancer Centres) described in this supplement is a significant accomplishment with several lessons for guideline developers around the world (Fervers et al, 2000).

The FNCLCC is a formal association with an administrative structure, including an administrative board consisting of the Directors of each of the 20 member independent Comprehensive Cancer Centres in France's public health system. The FNCLCC Centres have developed together programmatic initiatives that demonstrate the value of a cooperative and coordinated approach to addressing the cancer problem on a national scale. Each Centre contributes both financial and human resources, and they share clinical information. Such an initiative requires imaginative and dedicated leadership to keep the partners at the table for the common welfare of the system.

A programmatic approach to guideline development provides opportunities for addressing challenges that currently face the ad hoc nature of the guideline movement worldwide. Some of these challenges include: avoiding the duplication of effort where possible across guideline development groups; improving the connection between the development phase and the equally important 'aftercare phases' (maintenance, dissemination, implementation, evaluation and reporting) of guidelines; promoting bottom-up as opposed to the generally less effective top-down approach of many guideline development projects; improving opportunities for quality improvement and efficiency over time; suggesting opportunities for research collaborations.

### DUPLICATION OF EFFORT IN GUIDELINE DEVELOPMENT

Motivated by a desire to 'claim intellectual territory' and to maintain control over their own destinies, health professional societies have entered into the guideline development game. Many of these efforts began simultaneously, resulting in the production of guidelines that caused confusion in the field, especially when guidelines varied in quality and were inconsistent in their recommendations. This, combined with the huge effort and expense of developing an evidence-based guideline, has caused developers to realize the waste involved in ad hoc approaches.

Programmatic initiatives such as SOR provide the opportunity to think through guideline priorities and harness cooperation from a variety of sources, in order to develop their own documents, or adopt those of others', that are of high quality and acceptable to stakeholders. A programmatic approach provides the opportunity for a formal process of guideline 'adoption' or 'endorsement' by which programs would consider guidelines produced by other groups, and then set criteria for their use locally.

The process of endorsement/adoption begins during the literature searching phase of the development process, which ought to include the location of existing documents that might be relevant, thus avoiding the full-blown effort of starting from scratch. In this way, the generalizable features of already developed guidelines (i.e. the synthesized evidence) could be shared, with local groups developing recommendations that are unique to local interpretations of the evidence and consistent with local circumstances and values. Through such mechanisms, the overall costs of the systematic overviews (Mulrow, 1994) that are at the heart of evidence-based recommendations, can be reduced.

### LINKING DEVELOPMENT WITH IMPLEMENTATION AND EVALUATION

At the start, guideline efforts almost always underestimated the costs and effort required to develop documents that were truly 'evidence-informed' (Sackett et al, 1996) (a more appropriate term than 'evidence-based'). Ad hoc panels of otherwise busy volunteers were assembled to address clinical conditions comprehensively. By the time the guidelines were completed, the participants were eager to see them published, but had little interest in their 'aftercare'. Guideline 'aftercare' refers to the important activities of evidence updating (maintenance), dissemination, implementation, evaluation and reporting. Using the ad hoc approach, whatever financial resources were allocated to the project became quickly consumed during the development phase, leaving little available for aftercare.

The programmatic approach taken by SOR demonstrates the potential value of a balanced portfolio of aftercare activities that can be planned within predictable budgetary constraints. For example, the program could theoretically decide what proportion of its resources to use in any given year for the development of new guidelines, for maintenance of existing ones, or for evaluation (Ray-Coquard et al, 1997). The financial resources within SOR have become constrained, with additional funding needed for detailed implementation and evaluation. These are being planned to occur within regional networks.

A programmatic approach allows for strategic linkage between development and implementation phases. For SOR, part of the implementation strategy is built in at the start of the guideline development phase. Too often, guideline development, dissemination and implementation are conceptualized as sequential, as opposed to interacting phases along the path of a guideline's life-cycle.

### 'TOP-DOWN' VS 'BOTTOM-UP' GUIDELINE DEVELOPMENT STRATEGIES

While the SOR guideline development process adheres quite rigorously to 'evidence-informed' principles, it also respects experience through practitioner feedback and the use of expert consensus as equally legitimate inputs into clinical recommendations (Browman, 1999; Browman et al, 1999). The acknowledgement of the importance of blending clinical expertise with research evidence is an important step forward in advancing the evidence-informed approach as one that is practical and responsive. This approach is also more likely to achieve 'buy-in' from those who are expected to abide by the recommendations (Cabana et al, 1999). It is this 'bottom-up' feature of SOR that links implementation with development, and the success of this approach by SOR has been explicitly acknowledged by French health authorities.

### OPPORTUNITIES FOR QUALITY IMPROVEMENT AND EFFICIENCY OVER TIME

An important feature of the FNCLCC guideline development model is the use of a more or less permanent slate of panels, consisting of members who remain together for an extended time. This provides members with the opportunity to learn together so that their work improves over time in both quality and efficiency. Participation involves broad representation of expertise, including the guideline developers and expert reviewers from Cancer Centres, partner universities, general hospitals (all public sector) and private clinics. This enables regions to enhance dissemination of the message using opinion leaders who are part of the development process. As such, the process may serve as a valuable educational vehicle that can influence the entire culture of the cancer system.

Another important characteristic of the SOR model is the 'methodology resource group'. This enabling strategy makes guideline developers feel supported in their work, and also contributes to their continuing education as consumers and users of healthcare research. The methods resource group serves a quality-control function, to ensure that appropriate scientific methods are used to minimize bias in how the evidence is located, synthesized and interpreted, and it can also contribute to the maintenance phase of aftercare.

### OPPORTUNITIES FOR RESEARCH COLLABORATIONS

The programmatic nature of SOR positions it well to contribute to national and international research efforts to improve the guideline movement and the quality of guideline documents. Three aspects of the SOR/FNCLCC project demonstrate where such collaborations might be useful.

**i)** In 1994, Dr Beatrice Fervers of the FNCLCC visited McMaster University in Hamilton, Canada to review the methods and organization of the Cancer Care Ontario Clinical Practice Guideline Initiative (Browman et al, 1995; Evans et al, 1997). At that time, both initiatives were at a similar stage of evolution. Despite the programs having evolved independently in different countries, the program leaders noted remarkable parallels in the conceptualization, implementation and barriers associated with their approaches. This led to a comparative analysis of programs in different medical cultures from Canada and France, suggesting that an evidence-informed programmatic approach to guidelines within an identifiable cancer system may be generalizable (Fervers et al, 1997).

The collaboration has also provided insights into the legitimate reasons for inconsistency of guideline recommendations based on the same evidence, because of differences in national and medical cultural perspectives. One important cultural difference is the extensive involvement of lay people (community representatives) by the Ontario initiative, and their lack of representation in SOR.

**ii)** Each guideline produced by SOR is comprehensive in its attempt to cover all aspects of the management of a particular clinical condition. This is similar to national guidelines produced in Australia and New Zealand, but is in contrast with other programs, such as Cancer Care Ontario which uses guidelines to inform only narrow aspects of a condition, relating to a specific clinical decision of high priority. Canada's Steering Committee on Clinical Practice Guidelines for the Care and Treatment of Breast Cancer took a middle ground by producing ten specific guideline documents and then consolidating them as chapters within a more comprehensive document (Steering Committee on Clinical Practice Guidelines for the Care and Treatment of Breast Cancer, 1998a–k). Although there is value in a comprehensive approach, the key information and recommendations pertaining to specific decisions are often difficult to find without companion documents. SOR deals with this through the use of clinical algorithms as a practical guide for the clinician (Fervers et al, 1995). Furthermore, the updating process allows a focus on specific clinical questions of high priority that can be easily linked to the comprehensive document.

Also, guideline developers may have preferences as to whether they attack a problem comprehensively from the beginning or in more easily manageable priority chunks, and this may influence their motivation and work habits during the phase of development. Finally, there needs to be consideration of the relative value of clinical guidelines in areas where evidence is strong or weak. The objectives of individual guidelines that are designed to influence practice where evidence is strong may be strategically different from the objectives of guidelines intended to influence practice where evidence is weak. This can be a challenge for comprehensive guidelines because of the variation in quality of the evidence at different points along the management continuum of a disease. For the many situations where evidence may be weak, a programmatic approach can be used to implement formal consensus processes for developing recommendations that are credible.

**iii)** The use of 'levels of evidence' to communicate the quality of healthcare research results available to inform a clinical recommendation is a feature of virtually all 'evidence-informed' guideline processes (Cook et al, 1995). However, the application of this approach may suffer because it tends to be formulaic, and varies widely from one group to another.

The 'levels of evidence' construct was originally conceived as a form of descriptive shorthand. It was intended to convey, through categorization, the quality of evidence available, based on the rigor of the design of the research used to generate it.

*© 2001 FNCLCC*

The use of 'levels of evidence' is intended to convey the nature of *the best available* evidence that addresses a particular problem. For example, results from large randomized controlled trials of an intervention vs control for a given condition (level I evidence) trumps lower quality evidence, and it is therefore redundant to qualify a recommendation by more than one level of evidence as some guidelines do. But, even evidence categorized as of high quality (e.g. randomized controlled trials) can vary in quality depending on other design features of the research (e.g. blinding, concealment of allocation and adequacy of follow-up).

Levels of evidence have been used to grade the recommendations in guidelines. Such grades are intended to reflect the confidence with which a recommendation can be made. The SOR documents blend the traditional levels of evidence with grades of recommendations, which can create confusion. Our experience is that clinicians often have difficulty in making the correlation between a level of evidence and a recommendation grade. This is because of the variable quality of research evidence within levels, and the problem of generalizing the results of some trials of high quality.

The use of 'levels of evidence' as a shorthand descriptive tool was never intended to provide only positive support for recommendations about interventions. For example, evidence classified as poor (e.g. case reports or case series) is usually a signal that the evidence ought to be seriously challenged as a support for a positive recommendation. Yet many guideline developers (in their enthusiasm) use such low quality evidence as justification to recommend an intervention, as opposed to justifying its rejection. Some guideline development groups actually rate 'expert opinion' with a level of evidence. This may provide a veneer of rigor that does not actually exist, and reduces the usefulness of the 'levels of evidence' approach.

Some guideline programs have attached lower levels of evidence to recommendations, for the apparent sake of completeness. Often, such a recommendation (e.g. the use of a multidisciplinary approach, or the routine use of a complete blood count in initial patient evaluation) will never be tested in a trial, nor may it be worth testing, and it does not require slavish adherence to a formulaic framework.

These issues highlight the need for more research into how to appropriately use levels of evidence, how to link them, if at all, with clinical recommendations and how to report recommendations within a guideline. Programs like the SOR are well positioned to make valuable and insightful contributions to improving the overall quality of clinical guidelines, as an evolving healthcare technology for cancer (Browman et al, 1997). The achievements of the SOR program mark its investigators as potentially valuable contributors to international collaborations for improving the development and aftercare of clinical practice guidelines.

### REFERENCES

Browman GP (1999) Evidence-based paradigms and opinions in clinical management and cancer research. *Semin Oncol* **26**: 9–13

Browman GP, Newman TE, Mohide EA, Graham ID, Levine MN, Pritchard KI, Evans WK, Maroun JA, Hodson DI, Carey MS and Cowan DH (1998) Progress of clinical oncology guidelines development using the Practice Guidelines Development Cycle: the role of practitioner feedback. *J Clin Oncol* **16**: 1226–1231

Browman GP, Levine MN, Graham I, Whelan T, Sawka C, Pritchard KI, Jadad A and Newman TE (1997) The clinical practice guideline: an evolving health care technology. *Cancer Prev Control* **1**: 7–8

Browman GP, Levine MN, Mohide EA, Hayward RS, Pritchard KI, Gafni A and Laupacis A (1995) The practice guidelines development cycle: a conceptual tool for practice guidelines development and implementation. *J Clin Oncol* **13**: 502–512

Cabana MD, Rand CS, Powe NR, Wu AW, Wilson MH, Abboud PA and Rubin HR (1999) Why don't physicians follow clinical practice guidelines? A framework for improvement. *JAMA* **282**: 1458–1465

Cook DJ, Guyatt GH, Laupacis A, Sackett DL and Goldberg RJ (1995) Clinical recommendations using levels of evidence for antithrombotic agents. *Chest* **108**: 227S–230S

Evans WK, Newman T, Graham I, Rusthoven JJ, Logan D, Shepherd FA and Chamberlain D (1997) Lung cancer practice guidelines: lessons learned and issues addressed by the Ontario Lung Cancer Disease Site Group [see comments]. *J Clin Oncol* **15**: 3049–3059

Fervers B (2000) Les recommandations pour la pratique clinique: I'exemple des Standards, Options et Recommendations de la FNCLCC. In: Nouvelle évaluation médicale, Giraud A, Boissel JP, Fervers B, Grenier B, Launois R and Lepligen A (eds) Economica: Paris

Fervers B, Browman G, Newman T, Lehmann M, Sebban C and Philip T (1997) Clinical practice guidelines in oncology: two guidelines development programs in France and Canada. 2nd International Conference on Scientific Basis of Health Services, Amsterdam

Fervers B, Bonichon F, Demard F, Heron JF, Mathoulin S, Philip T, Renaud-Salis JL, Toma C and Bey P (1995) Methodology of the development of diagnostic and therapeutic standards, options and recommendations in oncology. *Bull Cancer* (Paris) **82**: 761–767

Mulrow CD (1994) Rationale for systematic reviews. *BMJ* **309**: 597–599

Ray-Coquard I, Philip T, Lehmann M, Fervers B, Farsi F and Chauvin F (1997) Impact of a clinical guidelines program for breast and colon cancer in a French cancer center. *JAMA* **278**: 1591–1595

Sackett DL, Rosenberg WM, Gray JA, Haynes RB and Richardson WS (1996) Evidence based medicine: what it is and what it isn't [editorial] [see comments]. *BMJ* **312**: 71–72

Steering Committee on Clinical Practice Guidelines for the Care and Treatment of Breast Cancer (1998a) The Steering Committee on Clinical Practice Guidelines for the Care and Treatment of Breast Cancer. *CMAJ* **158** (Suppl 3): S1–S2

Steering Committee on Clinical Practice Guidelines for the Care and Treatment of Breast Cancer (1998b) The palpable breast lump: information and recommendations to assist decision-making when a breast lump is detected. Canadian Association of Radiation Oncologists [see comments]. *CMAJ* **158** (Suppl 3): S3–S8

Steering Committee on Clinical Practice Guidelines for the Care and Treatment of Breast Cancer (1998c) Investigation of lesions detected by mammography. Canadian Association of Radiation Oncologists [see comments]. *CMAJ* **158** (Suppl 3): S9–14

Steering Committee on Clinical Practice Guidelines for the Care and Treatment of Breast Cancer (1998d) Mastectomy or lumpectomy? The choice of operation for clinical stages I and II breast cancer. Canadian Association of Radiation Oncologists [see comments]. *CMAJ* **158** (Suppl 3): S15–S21

Steering Committee on Clinical Practice Guidelines for the Care and Treatment of Breast Cancer (1998e) Axillary dissection. Canadian Association of Radiation Oncologists. *CMAJ* **158** (Suppl 3): S22–S26

Steering Committee on Clinical Practice Guidelines for the Care and Treatment of Breast Cancer (1998f) The management of ductal carcinoma in situ (DCIS). Canadian Association of Radiation Oncologists [see comments]. *CMAJ* **158** (Suppl 3): S27–S34

Steering Committee on Clinical Practice Guidelines for the Care and Treatment of Breast Cancer (1998g) Breast radiotherapy after breast-conserving surgery. Canadian Association of Radiation Oncologists. *CMAJ* **158** (Suppl 3): S35–S42

Steering Committee on Clinical Practice Guidelines for the Care and Treatment of Breast Cancer (1998h) Adjuvant systemic therapy for women with node-negative breast cancer. *CMAJ* **158** (Suppl 3): S43–S51

Steering Committee on Clinical Practice Guidelines for the Care and Treatment of Breast Cancer (1998i) Adjuvant systemic therapy for women with node-positive breast cancer. *CMAJ* **158** (Suppl 3): S52–S64

Steering Committee on Clinical Practice Guidelines for the Care and Treatment of Breast Cancer (1998j) Follow-up after treatment for breast cancer. *CMAJ* **158** (Suppl 3): S65–S70

Steering Committee on Clinical Practice Guidelines for the Care and Treatment of Breast Cancer (1998k) The management of chronic pain in patients with breast cancer. Canadian Society of Palliative Care Physicians. Canadian Association of Radiation Oncologists. *CMAJ* **158** (Suppl 3): S71–S81

© 2001 FNCLCC
